# Lack of Association of *TLR1* and *TLR5* Coding Variants with Mortality in a Large Multicenter Cohort of Melioidosis Patients

**DOI:** 10.4269/ajtmh.23-0381

**Published:** 2024-03-19

**Authors:** Thatcha Yimthin, Rungnapa Phunpang, Shelton W. Wright, Ekkachai Thiansukhon, Seksan Chaisuksant, Ploenchan Chetchotisakd, Kittisak Tanwisaid, Somchai Chuananont, Chumpol Morakot, Narongchai Sangsa, Wirayut Silakun, Sunee Chayangsu, Noppol Buasi, Ganjana Lertmemongkolchai, Narisara Chantratita, T. Eoin West

**Affiliations:** ^1^ Department of Microbiology and Immunology, Faculty of Tropical Medicine, Mahidol University, Bangkok, Thailand;; ^2^ Institute of Veterinary Bacteriology, Department of Infectious Diseases and Pathobiology, Vetsuisse Faculty, University of Bern, Bern, Switzerland;; ^3^ Graduate School for Cellular and Biomedical Sciences (GCB), University of Bern, Bern, Switzerland;; ^4^ Department of Pediatrics, University of Washington, Seattle, Washington;; ^5^ Department of Medicine, Udon Thani Hospital, Udon Thani, Thailand;; ^6^ Department of Medicine, Khon Kaen Regional Hospital, Khon Kaen, Thailand;; ^7^ Department of Medicine, Srinagarind Hospital, Khon Kaen University, Khon Kaen, Thailand;; ^8^ Department of Medicine, Nakhon Phanom Hospital, Nakhon Phanom, Thailand;; ^9^ Department of Medicine, Mukdahan Hospital, Mukdahan, Thailand;; ^10^ Department of Medicine, Roi Et Hospital, Roi Et, Thailand;; ^11^ Department of Medicine, Buriram Hospital, Buriram, Thailand;; ^12^ Department of Medicine, Surin Hospital, Surin, Thailand;; ^13^ Department of Medicine, Sisaket Hospital, Sisaket, Thailand;; ^14^ Department of Medical Technology, Faculty of Associated Medical Science, Chiang Mai University, Chiang Mai, Thailand;; ^15^ The Centre for Research and Development of Medical Diagnostic Laboratories, Khon Kaen University, Khon Kaen, Thailand;; ^16^ Mahidol-Oxford Tropical Medicine Research Unit, Faculty of Tropical Medicine, Mahidol University, Bangkok, Thailand;; ^17^ Department of Medicine, University of Washington, Seattle, Washington;; ^18^ Department Global Health, University of Washington, Seattle, Washington

## Abstract

Melioidosis, infection caused by *Burkholderia pseudomallei*, is characterized by robust innate immune responses. We have previously reported associations of *TLR1* single nucleotide missense variant rs76600635 with mortality and of *TLR5* nonsense variant rs5744168 with both bacteremia and mortality in single-center studies of patients with melioidosis in northeastern Thailand. The objective of this study was to externally validate the associations of rs76600635 and rs5744168 with bacteremia and mortality in a large multicenter cohort of melioidosis patients. We genotyped rs76600635 and rs5744168 in 1,338 melioidosis patients enrolled in a prospective parent cohort study conducted at nine hospitals in northeastern Thailand. The genotype frequencies of rs76600635 did not differ by bacteremia status (*P* = 0.27) or 28-day mortality (*P* = 0.84). The genotype frequencies of rs5744168 did not differ by either bacteremia status (*P* = 0.46) or 28-day mortality (*P* = 0.10). Assuming a dominant genetic model, there was no association of the rs76600635 variant with bacteremia (adjusted odds ratio [OR], 0.75; 95% CI, 0.54–1.04, *P* = 0.08) or 28-day mortality (adjusted OR, 0.96; 95% CI, 0.71–1.28, *P* = 0.77). There was no association of the rs5744168 variant with bacteremia (adjusted OR, 1.24; 95% CI, 0.76–2.03, *P* = 0.39) or 28-day mortality (adjusted OR, 1.22; 95% CI, 0.83–1.79, *P* = 0.21). There was also no association of either variant with 1-year mortality. We conclude that in a large multicenter cohort of patients hospitalized with melioidosis in northeastern Thailand, neither *TLR1* missense variant rs76600635 nor *TLR5* nonsense variant rs5744168 is associated with bacteremia or mortality.

## INTRODUCTION

Melioidosis is an infectious disease caused by *Burkholderia pseudomallei*, a flagellated Gram-negative bacterium found in tropical soil and water. This infection is of significant public health importance in southeast Asia and Australia and increasingly recognized as an emerging disease in other tropical regions. Clinical presentations of melioidosis range from acute sepsis to chronic and persistent infections, and the mortality rate can exceed 40% in areas where the disease is endemic.^1^ Various factors are associated with the outcome of this disease, such as age, underlying diseases, and clinical features of infection such as pneumonia or bacteremia.^2^ However, gaining further insights into risk factors for poor outcomes can advance knowledge about key biological mechanisms of the host response to melioidosis.

Studies of human genetic variation are powerful tools with which to understand susceptibility to infection and to outcomes from infection. For example, genome-wide studies have been applied to identify host genetic factors that affect susceptibility to infectious diseases such as tuberculosis, leprosy, enteric fever, and malaria.^3^^–^^6^ Recently, in Thai populations, polymorphisms in candidate innate immune genes, such as those encoding NOD2, tumor necrosis factor alpha (TNF-α), and Toll-like receptors (TLRs),^7^^–^^9^ have been found to be associated with susceptibility to melioidosis. Toll-like receptors are a family of receptors in the innate immune system that can recognize pathogen-associated molecular patterns from microorganisms and induce host inflammatory responses. Various TLRs have been shown to recognize components of *B. pseudomallei*.^10^ TLR1 heterodimerizes with TLR2 to mediate host responses to lipopeptides, peptidoglycan, and other parts of bacterial cell walls.^11^^,^^12^ Common genetic variation in *TLR1* has been associated with a higher risk of mortality in sepsis, more severe malaria, and *Helicobacter pylori* infection.^13^^–^^17^ In earlier work, we have shown that three common *TLR1* variants (rs5743551, rs4833095, and rs5743618) in Thais with melioidosis are not associated with death.^18^ However, we have reported that a novel coding variant in *TLR1* (rs76600635) is associated with a severe phenotype of bacteremic melioidosis in a discovery set and with mortality in a replication set of patients; this association with mortality persisted in a validation set of patients when differences in study design influencing timing of subject recruitment were taken into account.^19^ These observations implicate *TLR1* in the host response to melioidosis.

In addition, previous studies have suggested that TLR5, a receptor for bacterial flagellin, may be important in directing the host response to *B. pseudomallei*.^20^^–^^22^ We have found that the nonsense *TLR5* variant rs5744168 and the nonsynonymous *TLR5* variant rs5744174 are associated with impaired blood-derived inflammatory cytokine responses to flagellin.^20^^,^^23^ We have also shown, in two separate studies, that melioidosis patients carrying the *TLR5* variant rs5744168 are protected from organ failure and have improved survival.^23^^,^^24^ Patients with rs5744168 are also less likely to be bacteremic.^24^ These studies suggest that *TLR5* plays a significant role in modulating host defenses during melioidosis.

A limitation to our earlier genetic association studies in melioidosis, however, is that they have all been performed at a single center. Validation in independent cohorts of melioidosis patients is necessary to confirm the observed associations. Therefore, in this study, we take advantage of a large prospective, multicenter observational study that recruited melioidosis patients hospitalized in northeastern Thailand to test the associations of *TLR1* variant rs76600635 and *TLR5* variant rs5744168 with bacteremia and mortality in melioidosis.

## MATERIALS AND METHODS

### Study design and patients.

The study cohort was composed of melioidosis patients who were admitted to nine hospitals in northeastern Thailand from July 2015 to December 2018 and enrolled in an observational study that has been described previously.^2^ Patients at least 15 years of age with microbiologically confirmed melioidosis met enrollment criteria. Pregnancy, receipt of palliative care, or incarceration were exclusion criteria. The diagnosis of melioidosis was determined by culture of an organism that was identified as *B. pseudomallei* by standard methodologies and/or Vitek 2, a positive latex agglutination test, or immunofluorescence microscopy assay.^25^^–^^27^ To reduce confounding by population differences, for this study we analyzed patients who reported their race as Asian.

### Sample collection.

Four milliliters of whole blood was collected from enrolled patients into ethylenediaminetetraacetic acid blood tubes (BD, Franklin Lakes, NJ). These samples were centrifuged at 1,500 × *g* for 10 min, and packed cell samples (containing buffy coat and red blood cells) were collected and stored at –20°C or –80°C at the hospitals. The frozen samples were transported on dry ice with a temperature recorder to the laboratory in Bangkok for DNA extraction.

### DNA extraction and genotyping.

DNA was extracted from 1 mL of packed cells using the QIAamp DNA blood midi kit (Qiagen, Hilden, Germany) according to the manufacturer’s instructions. DNA concentration and purity were determined at by A260/280 and A260/230 ratios, respectively, using a NanoDrop spectrophotometer (Thermo Fisher Scientific, Waltham, MA) and examination by 0.8% agarose gel electrophoresis. Genotyping of *TLR1* variant rs76600635 (130A/G) and *TLR5* variant rs5744168 (1174C/T) was performed using a TaqMan single nucleotide polymorphism genotyping assay (Applied Biosystems, Foster City, CA). The cycle conditions were as follows: one cycle of 95°C for 30 seconds, followed by 40 cycles of 95°C for 15 seconds and 65°C for 45 seconds. Real-time polymerase chain reaction (PCR) was performed on a CFX96 Touch^TM^ real-time PCR detection system (Bio-Rad, Hercules, CA). Variant calling was made automatically using Bio-Rad CFX Manager^TM^ v. 3.0 software. The amplification curves were checked for each genotype manually for final allele discrimination and also confirmed by DNA sequencing.

### Exposures and outcomes.

Exposures were defined as the *TLR1* rs76600635 and *TLR5* rs5744168 variant genotypes. Two primary outcomes were analyzed: bacteremia and mortality 28 days after study enrollment. Bacteremia was defined as a positive blood culture for *B. pseudomallei* among the subset of patients who had blood cultures drawn. One secondary outcome was also analyzed: mortality 1 year after study enrollment.

### Power and statistical analysis.

Power was estimated using the “genpwr” package in R based on minor allele frequencies (MAFs) and odds ratios (ORs) of effect observed in our prior studies^19^^,^^23^^,^^24^ and on the size and frequency of outcomes in the present patient cohort. For *TLR5* rs5744168, with an expected MAF of 0.05 to 0.10, the power to detect ORs of 0.2 to 0.5 for mortality and bacteremia (at rates of 25% and 82%, respectively, in the present cohort) was 0.83 to 1.00, with alpha set at 0.05 and assuming a dominant model. For *TLR1* rs76600635, with an expected MAF of 0.13 to 0.20, the power to detect ORs of 1.5 to 4.0 for mortality was 0.81 to 1.00 and for bacteremia was 0.61 to 1.00, with alpha set at 0.05 and assuming a dominant model. Variants were tested for departure from the Hardy-Weinberg equilibrium by use of the exact test. Association of genotypes with bacteremia or mortality was performed using the exact test. Crude and adjusted logistic regression, assuming a dominant model (in line with our past studies), was performed. The covariates included were age, sex, comorbidities, site, referral status, and time from admission to study enrollment.^19^^,^^23^^,^^24^ Results were expressed as ORs, with corresponding 95% CI. A *P*-value of <0.05 was defined as statistically significant. All statistical analyses were performed with Stata SE v. 17.0 (Stata Corp, College Station, TX).

### Ethics statement.

Written informed consent was obtained from all participants or their representatives. The study was approved by the ethical committees of the Faculty of Tropical Medicine, Mahidol University, Udon Thani Hospital, Khon Kaen Hospital, Srinagarind Hospital, Nakhon Phanom Hospital, Mukdahan Hospital, Roi Et Hospital, Buriram Hospital, Surin Hospital, and Sisaket Hospital. The University of Washington issued a statement of nonengagement in human subject research.

## RESULTS

Of 1,345 patients enrolled in the parent study for whom the 28-day mortality outcome was known, DNA was not available for six and one identified as non-Asian; therefore, a total of 1,338 melioidosis patients were analyzed. Of these, 335 (25.0%) died. Blood cultures were obtained for 1,254 of the patients, and 1,032 (82.3%) were bacteremic with *B. pseudomallei*. The characteristics of the study population are given in Table 1. Of all patients, the median age was 55 years old (interquartile range [IQR], 46–64). Most patients (966, 72.1%) were male. The most common comorbidity was diabetes (941, 70.3%).

**Table 1:** Selected demographic and clinical characteristics of melioidosis patients studied

Patient Characteristics	All (*N* = 1,338)	Bacteremia		28-Day Mortality	
		Yes (*n* = 1,032)	No (*n* = 222)	Died (*n* = 335)	Survived (*n* = 1,003)
Age in years, median (IQR)	55 (46–64)	55 (46–64)	56 (47–65)	57 (47–66)	54 (46–63)
Male sex, *n* (%)	966 (72.1)	742 (71.9)	169 (76.1)	253 (75.5)	712 (71.0)
Diabetes, *n* (%)	941 (70.3)	745 (72.2)	144 (64.9)	220 (65.7)	720 (71.8)
Chronic kidney disease, *n* (%)	228 (17.0)	184 (17.8)	31 (14.0)	72 (21.5)	156 (15.6)
Chronic lung disease, *n* (%)	163 (12.2)	114 (11.1)	42 (18.9)	58 (17.3)	104 (10.4)
Heart disease, *n* (%)	62 (4.6)	48 (4.7)	11 (5.0)	23 (6.9)	38 (3.8)
Chronic liver disease, *n* (%)	47 (3.5)	42 (4.1)	5 (2.3)	23 (6.9)	24 (2.4)

IQR = interquartile range.

The MAFs of *TLR1* rs76600635 and *TLR5* rs5744168 in the entire cohort were 0.15 and 0.06, respectively, similar to the MAFs we have previously reported in melioidosis patients in northeast Thailand.^19^^,^^23^^,^^24^ For both variants, we determined that there was no deviation from Hardy-Weinberg equilibrium in the nonbacteremic patients or in the nonfatal cases (all *P* >0.05). The genotype distributions of rs76600635 and rs5744168 in melioidosis patients according to bacteremia and mortality are shown in Table 2. There were no significant differences in frequency of bacteremia or 28-day mortality by genotype of either variant.

**Table 2:** Distribution of rs76600635 and rs5744168 genotypes among patients by bacteremia and 28-day mortality

Genotype	Bacteremia (number [%] of patients)		*P*-Value*	28-Day Mortality (number [%] of patients)		*P*-Value*
	Yes	No		Died	Survived	
rs76600635						
AA	766 (74.2)	154 (69.4)	0.27	247 (73.7)	722 (72.0)	0.84
AG	252 (24.4)	64 (28.8)		83 (24.8)	266 (26.5)	
GG	14 (1.4)	4 (1.8)		5 (1.5)	15 (1.5)	
rs5744168						
CC	905 (87.7)	199 (89.6)	0.46	290 (86.6)	888 (88.5)	0.10
CT	121 (11.7)	21 (9.5)		40 (11.9)	111 (11.1)	
TT	6 (0.6)	2 (0.9)		5 (1.5)	4 (0.4)	

* *P*-values were determined by the exact test.

We next performed logistic regression of the association of rs76600635 with bacteremia, assuming a dominant model (Table 3), and did not find that the variant was associated with bacteremia in an unadjusted model or after adjustment for covariates including age, sex, comorbidities, site, referral status, and time to enrollment (adjusted OR, 0.75; 95% CI, 0.54–1.04, *P* = 0.08). Similarly, there were no associations between rs5744168 and bacteremia (adjusted OR, 1.24; 95% CI, 0.76–2.03, *P* = 0.39). There was no association between either variant and 28-day mortality (rs76600635: adjusted OR, 0.96; 95% CI, 0.71–1.28, *P* = 0.77; rs5744168: adjusted OR, 1.22; 95% CI, 0.83–1.79, *P* = 0.32).

**Table 3:** Odds of bacteremia or 28-day mortality for carriers of rs76600635 or rs5744168 variants, assuming a dominant genetic model

Parameter	Variant	Unadjusted			Adjusted*		
		OR	95% CI	*P*-Value	OR	95% CI	*P*-Value
Bacteremia	rs76600635	0.79	0.57–1.08	0.14	0.75	0.54–1.04	0.08
	rs5744168	1.21	0.76–1.94	0.42	1.24	0.76–2.03	0.39
28-Day mortality	rs76600635	0.92	0.69–1.21	0.54	0.96	0.71–1.28	0.77
	rs5744168	1.20	0.83–1.73	0.34	1.22	0.83–1.79	0.32

OR = odds ratio.

* For adjusted model, age, sex, comorbidities, site, referral status, and time to enrollment were included as covariates.

A notable feature of our melioidosis cohort was that patients were followed for 1 year after study enrollment. Conceivably, carriers of variants in *TLR1* or *TLR5* may be at risk for delayed complications. We therefore tested the association of each variant with 1-year mortality in the 1,315 individuals for which this outcome was known. Examining genotype distributions (Table 4) or odds of death for variant carriers assuming a dominant genetic model, we found no associations with 1-year mortality (rs76600635: adjusted OR, 0.94; 95% CI, 0.71–1.23, *P* = 0.64; rs5744168: adjusted OR, 1.16; 95% CI, 0.81–1.67, *P* = 0.42).

**Table 4:** Distribution of rs76600635 and rs5744168 genotypes among patients by 1-year mortality

Genotype	1-Year Mortality (number [%] of patients)		*P*-Value*
	Died	Survived	
rs76600635			
AA	332 (74.4)	618 (71.1)	0.41
AG	109 (24.4)	237 (27.3)	
GG	5 (1.1)	14 (1.6)	
rs5744168			
CC	387 (86.8)	770 (88.6)	0.29
CT	54 (12.1)	95 (10.9)	
TT	5 (1.1)	4 (0.5)	

* *P*-values were determined by the exact test.

## DISCUSSION

In this study, we evaluated two innate immune single nucleotide genetic variants that we have found to be associated with bacteremia and/or death in melioidosis patients in single-center studies.^19^^,^^23^^,^^24^ However, in this larger analysis of a multicenter cohort of melioidosis patients, we did not replicate our previous findings. These results underscore the importance of external validation of genetic associations with clinical phenotypes, something that was not possible until recently in large cohorts of melioidosis patients.

The MAF of rs76600635, investigated by the 1000 Genomes Project Phase 3 in Ensembl, varies from 0.04 to 0.13 among five populations in East Asia, whereas this variant has not been found in African, American, or European populations. However, our past studies and the current investigation show that the MAF is higher (∼0.15) in northeastern Thai populations.^19^ rs76600635 represents a serine-to-proline amino acid substitution at position 44 in the *TLR1* gene on chromosome 4 and is predicted by PolyPhen-2 to be benign.^28^ In one functional study, rs76600635 does not alter NF-kB activation.^29^ However, the variant has been associated with thrombocytopenia in western Chinese patients who received antituberculosis drugs.^19^^,^^30^

rs5744168 results in the replacement of arginine at position 392 with a premature stop codon in the *TLR5* gene on chromosome 1.^31^ This leads to impaired flagellin-dependent and flagellin-independent signaling.^23^^,^^31^^,^^32^ The MAF of this variant is 0.02 to 0.10 worldwide, and our previous and current studies indicate that the MAF in northeastern Thai populations is 0.06 to 0.07.^23^^,^^24^ This variant has been associated with a number of other infectious and noninfectious clinical phenotypes.^31^^,^^33^^,^^34^

There are several possible explanations why the findings in this report are discordant with our previous publications. First, our original observations (although, for mortality, internally validated at a single center) may simply have been due to chance. Second, despite the larger size of the present cohort and MAFs comparable to those of our earlier cohorts, the current study may nonetheless lack sufficient power to detect true associations. Third, there may be unmeasured confounders of the association of genotype and clinical phenotype. In particular, there may be differential population stratification. Fourth, differences in study design, such as timing of patient enrollment, may contribute. Lastly, differences in clinical management over time or at different hospitals may play a role.

The major strengths of this analysis are the large sample size and the recruitment of melioidosis patients from multiple centers. In fact, this is the largest study of host genetic variation in melioidosis undertaken to date. However, there are some limitations to our study. We did not recruit patients who died early after admission to hospital, as culture-proven melioidosis was an enrollment requirement (although this approach was similar to several of our previous studies). In addition, there was a range of time from admission to enrollment. A number of patients did not have blood cultures obtained and therefore were excluded from the analyses of genotype and bacteremia.

In conclusion, *TLR1* variant rs76600635 and *TLR5* variant rs5744168 are not associated with bacteremia or mortality in melioidosis patients in a large, prospective, multicenter cohort study in northeastern Thailand. These results do not support our previous findings performed at a single center and highlight the importance of external validation of the association of human genetic variants with disease characteristics and outcomes.
